# Proteomic analysis reveals activation of platelet- and fibrosis-related pathways in hearts of *ApoE*^−/−^ mice exposed to diesel exhaust particles

**DOI:** 10.1038/s41598-023-49790-y

**Published:** 2023-12-19

**Authors:** Inkyo Jung, Yoon Jin Cho, Minhan Park, Kihong Park, Seung Hee Lee, Won-Ho Kim, Hyuk Jeong, Ji Eun Lee, Geun-Young Kim

**Affiliations:** 1 Division of Cardiovascular Disease Research, Department of Chronic Disease Convergence Research, Korea National Institute of Health, 187 Osongsaengmyeng2-ro, Osong-eub, Heungdeok-gu, Cheongju-si, Chungcheongbuk-do 28159 Republic of Korea; 2 Chemical and Biological Integrative Research Center, Biomedical Research Division, Korea Institute of Science and Technology, 5, Hwarang-ro 14-gil, Seongbuk-gu, Seoul, 02792 Republic of Korea; 3 Department of Chemistry, Sookmyung Women’s University, Cheongpa-ro 47-gil 100, Yongsan-gu, Seoul, 04310 Republic of Korea; 4 School of Earth Science and Environmental Engineering, Gwangju Institute of Science and Technology, 123 Cheomdangwagi-ro, Buk-gu, Gwangju, 61005 Republic of Korea

**Keywords:** Environmental sciences, Diseases

## Abstract

Air pollution is an environmental risk factor linked to multiple human diseases including cardiovascular diseases (CVDs). While particulate matter (PM) emitted by diesel exhaust damages multiple organ systems, heart disease is one of the most severe pathologies affected by PM. However, the in vivo effects of diesel exhaust particles (DEP) on the heart and the molecular mechanisms of DEP-induced heart dysfunction have not been investigated. In the current study, we attempted to identify the proteomic signatures of heart fibrosis caused by diesel exhaust particles (DEP) in CVDs-prone apolipoprotein E knockout (*ApoE*^*−/−*^) mice model using tandem mass tag (TMT)-based quantitative proteomic analysis. DEP exposure induced mild heart fibrosis in *ApoE*^*−/−*^ mice compared with severe heart fibrosis in *ApoE*^*−/−*^ mice that were treated with CVDs-inducing peptide, angiotensin II. TMT-based quantitative proteomic analysis of heart tissues between PBS- and DEP-treated *ApoE*^*−/−*^ mice revealed significant upregulation of proteins associated with platelet activation and TGFβ-dependent pathways. Our data suggest that DEP exposure could induce heart fibrosis, potentially via platelet-related pathways and TGFβ induction, causing cardiac fibrosis and dysfunction.

## Introduction

Air pollution is a heterogeneous mixture of particles and gases^1^. It is an important environmental risk factor linked to human diseases such as ischemic heart disease, cerebrovascular disease, lung cancer, acute lower respiratory disease, and chronic obstructive pulmonary disease^2^. The adverse effects of air pollution are primarily attributed to particulate matter with diameter < 2.5 μm (PM2.5). Global PM2.5-related mortality was estimated to be 3.16 million in 2010 and is predicted to cause 5.17 million deaths in 2050^3^. Interestingly, the majority of mortality in 2050 is predicted to be related to ischemic heart disease (2.17 million) and cerebrovascular disease (2.60 million)^3^, underscoring the importance of investigating the effects of PM2.5-induced cardiovascular diseases (CVDs) and developing therapeutic interventions.

Diesel exhaust particles (DEP), produced from combustion of diesel fuel, enter the human body via inhalation^4^. Although coarse DEP are trapped in airways and lungs, small particles (diameter < 2.5 μm) penetrate the respiratory system, circulate in blood vessels, and cause dysfunction of multiple organs, including the heart^5–7^. Previous studies have identified that thrombosis contributes to DEP-induced CVDs^8–11^, but the regulatory mechanisms for these pathologies remain incompletely understood.

Because PM is heterogenous, its components differ depending on the location of collection^4^. It is therefore not feasible to obtain environmental PM of reliable consistency to investigate its adverse effects on human disease. To compensate for the heterogenous makeup of PM, we combusted gasoline fuel or diesel fuel in the laboratory and produced consistent gasoline exhaust particles (GEP) and DEP with consistent makeup^12,13^. We previously analyzed GEP components and identified altered gene expression patterns in human umbilical vein endothelial cells (HUVECs) exposed to GEP^12^. Furthermore, after analyzing the components of DEP, we investigated their adverse effects on HUVECs, identifying that accumulation of autophagosomes induces HUVECs apoptosis by impairing autophagic flux and activating the caspase-8-caspase-3 cascade^13^. However, the in vivo effects of DEP on the heart and their related molecular mechanisms of potential DEP-induced heart dysfunction have not been investigated.

To determine how DEP affected cardiac fibrosis and protein levels, we exposed apolipoprotein E knockout (*ApoE*^*−/−*^) mice to DEP with intratracheal instillation and investigated changes of heart tissue protein expression patterns using tandem mass tag (TMT) labeling followed by liquid chromatography-tandem mass spectrometry (LC–MS/MS), allowing simultaneous comparison of protein abundances in all heart tissues. We chose *ApoE*^*−/−*^ mouse model because ApoE is an essential component of lipoprotein which plays a role in uptake of chylomicrons and very low-density lipoprotein (VLDL) and serves as a ligand to hepatic receptors such as LDL receptor (LDLR) for the cholesterol clearance^14,15^. ApoE and LDLR interplay was important in the regulation of cholesterol metabolism^16^, thus the absence of ApoE delayed lipoprotein clearance and developed hyperlipoproteinemia^17^, leading to CVDs evidenced by previous studies with *ApoE*^*−/−*^ mouse model^14,18,19^. DEP exposure induced mild heart fibrosis in *ApoE*^*−/−*^ mice compared with severe heart fibrosis in AngII-infused *ApoE*^*−/−*^ mice, and the most highly upregulated protein by DEP exposure was platelet factor 4 (PF4). Subsequently, we performed gene ontology (GO) analysis of proteins upregulated by DEP exposure, identifying that platelet activation is the primary process induced by DEP. Ingenuity pathways analysis (IPA) revealed that pathways related to CVDs and cardiac dysfunction were the significantly affected disease and toxicity phenotypes. Further, network analysis identified transforming growth factor β (TGFβ) as a major hub for signaling pathways regulated by DEP. These data suggested that DEP exposure induced heart fibrosis, potentially via platelet-related pathways and TGFβ induction, causing cardiac fibrosis and dysfunction.

## Results

### DEP exposure induced heart fibrosis in *ApoE*^*−/−*^ mice

**Figure 1:**
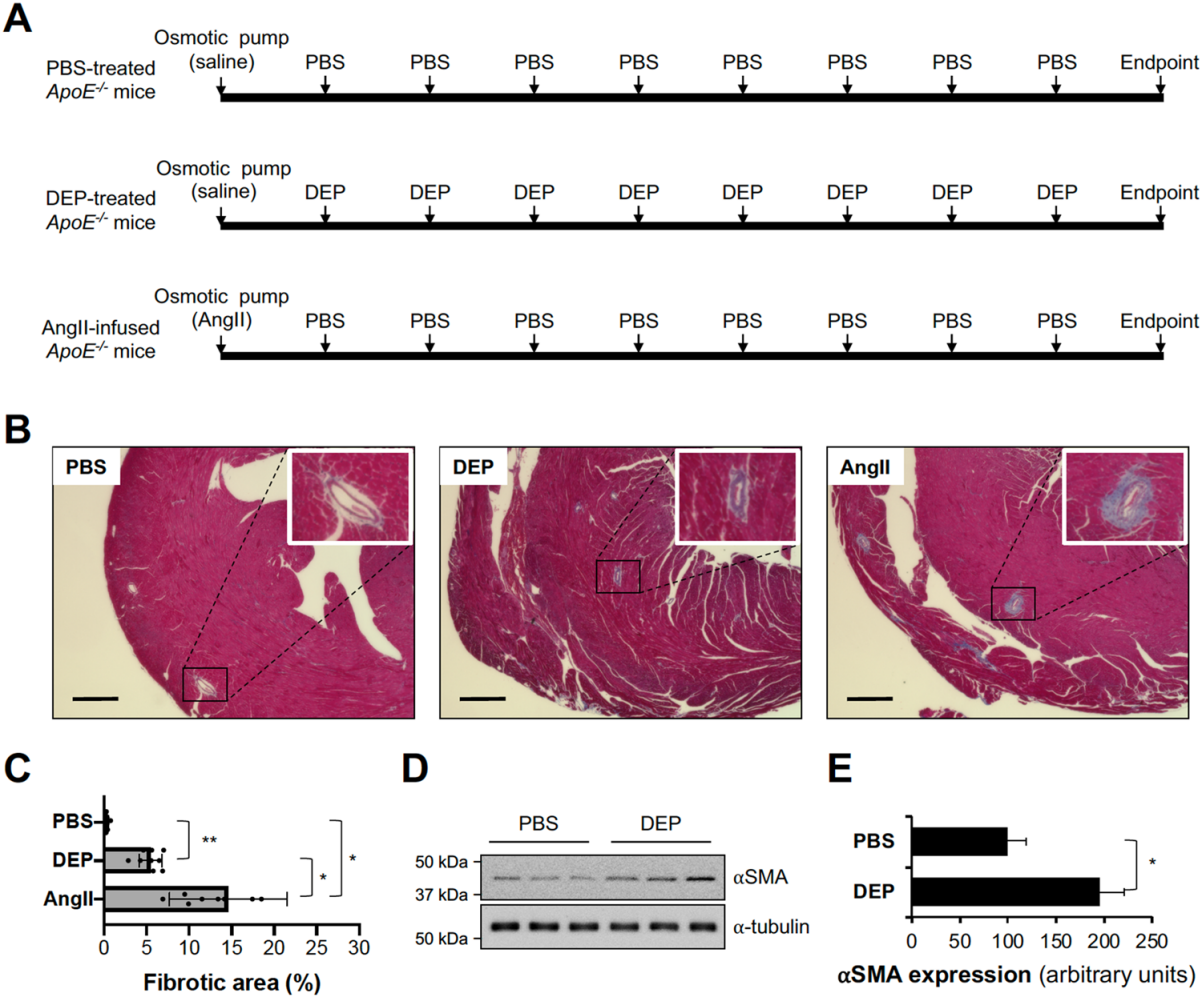
Heart fibrosis in *ApoE*^*−/−*^ mice exposed to DEP. (**A**) Schematic illustration of DEP exposure and AngII infusion protocol. *ApoE*^*−/−*^ mice were implanted with osmotic pumps containing saline or AngII, and PBS or extracted DEP (100 µg) that included soluble components in PBS and particles smaller than 0.2 µm was administered eight times total in 3 day intervals via intratracheal instillation. Three days following the final DEP treatment, mice were euthanized, and hearts were harvested. (**B**) Representative images of Masson’s trichrome staining in PBS-treated, DEP-treated, and AngII-infused hearts of *ApoE*^*−/−*^ mice. Scale bar, 400 µm. (**C**) The fibrotic area of each image was quantified using Image J software. n = 9. Results are presented as means ± SD. Statistical analysis was performed using the one-way ANOVA and Tukey’s post hoc test. *P < 0.02, **P < 0.05. (**D**) Western blotting was used to measure protein levels of the fibrosis markers ⍺SMA and ⍺-tubulin in hearts of *ApoE*^*−/−*^ mice treated with PBS or DEP. Membranes were cut prior to hybridization with antibodies. While the cropped images are seen, uncropped blots are provided in Supplementary Information. (**E**) Quantification of ⍺SMA protein levels normalized to ⍺-tubulin. Results are presented as means ± SD. *P < 0.02, two-tailed Student’s *t* test.

We established a mouse model of intratracheal DEP instillation to study potential heart damage. Originally, heart fibrosis was compared in WT mice subjected to intratracheal administration of PBS or DEP (Figure S1A). However, DEP did not induce heart fibrosis in WT mice, as demonstrated by Masson’s trichrome staining (Figure S1B-C). Thus, we used CVDs-prone *ApoE*^*−/−*^ mice to evaluate the potential adverse effect of DEP on animals with a predisposition for heart disease (Fig. 1A). When we performed this study, we used the angiotensin II (AngII)-induced heart damage model in *ApoE*^*−/−*^ mice as a positive control (Fig. 1A) to compare the severity of heart fibrosis in the positive control with that induced by DEP exposure. After implantation of an osmotic pump containing saline or AngII, intratracheal administration of DEP or PBS was performed eight times (every 3 day over 25 days). Three days following the final intratracheal administration, mice were sacrificed. Subsequently, hearts were harvested, and fibrosis and expression of proteins related to the fibrosis were examined. Masson’s trichrome staining of *ApoE*^*−/−*^ mice hearts revealed that DEP exposure induced mild fibrosis compared with severe fibrosis induced by AngII infusion (Fig. 1B, C). The finding of fibrosis in DEP-exposed *ApoE*^*−/−*^ mice hearts was supported by increased expression of ⍺-smooth muscle actin (⍺SMA), a key protein upregulated in heart fibrosis (Fig. 1D, E)^20,21^.

### TMT-based quantitative proteomic analysis

**Figure 2:**
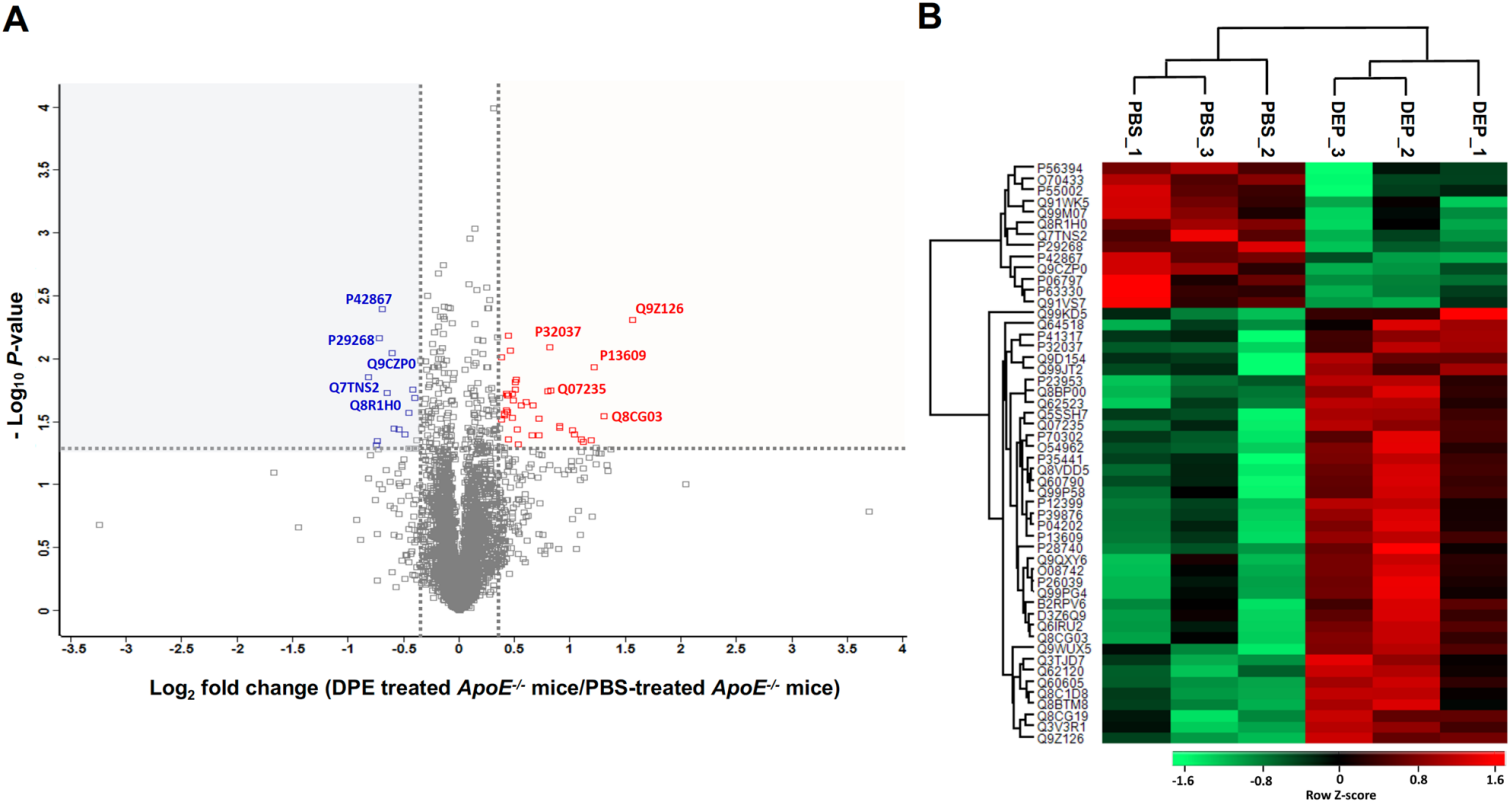
Differential heart protein expression pattern in *ApoE*^*−/−*^ mice exposed to DEP. (**A**) Volcano plot constructed from TMT labeling-based quantification data of the 5,038 identified proteins according to the statistical *P*-value (−log_10_*P*-value as y-axis) and relative abundance ratio (log_2_fold change as x-axis) between PBS-treated and DEP-treated *ApoE*^*−/−*^ mice. Thirty-nine proteins were significantly increased (*P*-value < 0.05, > 1.3-fold), and 13 proteins were significantly decreased (*P*-value < 0.05, > 1.3-fold) in DEP-treated *ApoE*^*−/−*^ mice. Upregulated proteins are denoted in red, and downregulated proteins are denoted in blue. UniProt accession numbers are also specified for the top five proteins exhibiting significant increases and decreases in DEP-treated *ApoE*^*−/−*^ mice. (**B**) Heat map demonstrating hierarchical clustering of 52 proteins with statistically significant changes (*P*-value < 0.05, > 1.3-fold) between PBS-treated and DEP-treated *ApoE*^*−/−*^ mice. The rows represent each individual protein, and the columns represent the three biological replicates of PBS-treated and DEP-treated *ApoE*^*−/−*^ mice. Hierarchical clustering of the 52 proteins was performed on log-transformed normalized abundance values after z-score normalization of the data using Perseus software (1.6.14.0).

We prepared heart tissue lysates from five groups consisting of PBS- or DEP-treated WT mice, PBS- or DEP-treated *ApoE*^*−/−*^ mice, and AngII-infused *ApoE*^*−/−*^ mice for proteomic analysis (n = 3 mice/group). From TMT labeling followed by LC–MS/MS analysis, 6,518 total proteins were identified from the five groups of mouse samples, and 6,461 proteins were commonly identified in the five groups (Table S1 and Figure S2). Spectra with an average reporter signal-to-noise ratio threshold ≥ 10 across 16 TMTpro 16plex channels were considered for quantification, with 5,038 proteins subjected to quantitative analysis (Table S2). To identify proteins that were significantly changed by DEP exposure in WT and *ApoE*^*−/−*^ mice, a Student’s *t* test comparison of the log_2_(normalized signal-to-noise ratio) values was conducted. Statistical analysis identified that 73 proteins were significantly different (*P*-value < 0.05) between PBS- and DEP-treated WT mice, and 235 proteins were significantly different (*P*-value < 0.05) between PBS- and DEP-treated *ApoE*^*−/−*^ mice (Tables S3 and S4). Using the criteria of |fold change|> 1.3 and *P*-value < 0.05, 17 proteins were differentially expressed between PBS- and DEP-treated WT mice (Table S5), and 52 proteins showed statistically significant changes between PBS- and DEP-treated *ApoE*^*−/−*^ mice (Fig. 2). More proteins were significantly changed by DEP exposure in *ApoE*^*−/−*^ mice than in WT mice, suggesting that the *ApoE*^*−/−*^ mouse model was potentially more susceptible to DEP exposure. In hearts of DEP-exposed *ApoE*^*−/−*^ mice, 39 proteins were upregulated, and 13 proteins were downregulated (Fig. 2A). A hierarchical clustering analysis of the 52 proteins revealed that the expression patterns between PBS- and DEP-treated *ApoE*^*−/−*^ mice were distinct (Fig. 2B). The 39 upregulated proteins in hearts of DEP-treated *ApoE*^*−/−*^ mice are listed in Table 1. The most highly upregulated protein was platelet factor 4 (PF4, Q9Z126) followed by cGMP-specific 3',5'-cyclic phosphodiesterase (Q8CG03), serglycin (SRGN, P13609), and ras-related protein Rab-27B (Q99P58). We also analyzed protein expression pattern in hearts of AngII-infused *ApoE*^*−/−*^ mice versus those of PBS-treated *ApoE*^*−/−*^ mice, and the abundance of 438 proteins was significantly different (*P*-value < 0.05) (Table S6). Using the criteria of |fold change|> 1.3 and *P*-value < 0.05 between groups, 143 proteins were differentially expressed following AngII infusion in *ApoE*^*−/−*^ mice, with 118 upregulated proteins (Table 2). Among upregulated proteins, five proteins ((inositol 1,4,5-triphosphate receptor associated 1 (Q9WUX5), zyxin (Q62523), protein IWS1 homolog (Q8C1D8), latent-transforming growth factor beta-binding protein 1 (Q8CG19), and carboxylesterase 1C (P23953)) were upregulated by both DEP exposure and AngII infusion in *ApoE*^*−/−*^ mice.

**Table 1:** Proteins upregulated by DEP exposure in hearts of *ApoE*^*−/−*^ mice.

Accession no	Gene symbol	Protein description	Fold change	*P*-value
Q9Z126	PF4	Platelet factor 4	2.94	0.005
Q8CG03	PDE5A	cGMP-specific 3',5'-cyclic phosphodiesterase	2.41	0.029
P13609	SRGN	Serglycin	2.30	0.012
Q99P58	RAB27B	Ras-related protein Rab-27B	2.20	0.044
Q99PG4	RGS18	Regulator of G-protein signaling 18	2.19	0.046
D3Z6Q9	BIN2	Bridging integrator 2	2.10	0.044
B2RPV6	MMRN1	Multimerin-1	2.02	0.040
P35441	THBS1	Thrombospondin-1	1.98	0.037
P04202	TGFB1	Transforming growth factor beta-1 proprotein	1.88	0.034
O08742	GP5	Platelet glycoprotein V	1.87	0.035
P32037	SLC2A3	Solute carrier family 2, facilitated glucose transporter member 3	1.77	0.008
Q07235	SERPINE2	Glia-derived nexin	1.75	0.018
P12399	CTLA2A	Protein CTLA-2-alpha	1.75	0.018
Q8BTM8	FLNA	Filamin-A	1.67	0.030
P41317	MBL2	Mannose-binding protein C	1.62	0.041
P39876	TIMP3	Metalloproteinase inhibitor 3	1.59	0.024
Q60790	RASA3	Ras GTPase-activating protein 3	1.57	0.040
Q9WUX5	IRAG1	Inositol 1,4,5-triphosphate receptor associated 1	1.50	0.022
Q64518	ATP2A3	Sarcoplasmic/endoplasmic reticulum calcium ATPase 3	1.49	0.023
P26039	TLN1	Talin-1	1.44	0.036
Q9QXY6	EHD3	EH domain-containing protein 3	1.43	0.048
Q62523	ZYX	Zyxin	1.42	0.015
Q6IRU2	TPM4	Tropomyosin alpha-4 chain	1.42	0.015
Q3V3R1	MTHFD1L	Monofunctional C1-tetrahydrofolate synthase, mitochondrial	1.42	0.018
Q8C1D8	IWS1	Protein IWS1 homolog	1.41	0.022
O54962	BANF1	Barrier-to-autointegration factor	1.40	0.019
Q9D154	SERPINB1A	Leukocyte elastase inhibitor A	1.39	0.030
Q8BP00	IQCB1	IQ calmodulin-binding motif-containing protein 1	1.39	0.009
Q99KD5	UNC45A	Protein unc-45 homolog A	1.37	0.044
Q60605	MYL6	Myosin light polypeptide 6	1.36	0.007
Q8CG19	LTBP1	Latent-transforming growth factor beta-binding protein 1	1.36	0.020
Q8VDD5	MYH9	Myosin-9	1.35	0.026
Q62120	JAK2	Tyrosine-protein kinase JAK2	1.35	0.019
Q5SSH7	ZZEF1	Zinc finger ZZ-type and EF-hand domain-containing protein 1	1.35	0.020
P70302	STIM1	Stromal interaction molecule 1	1.35	0.026
Q3TJD7	PDLIM7	PDZ and LIM domain protein 7	1.33	0.027
Q99JT2	STK26	Serine/threonine-protein kinase 26	1.32	0.028
P28740	KIF2A	Kinesin-like protein KIF2A	1.31	0.030
P23953	CES1C	Carboxylesterase 1C	1.30	0.010

**Table 2:** Proteins upregulated by AngII infusion in hearts of *ApoE*^*−/−*^ mice.

Accession no	Gene symbol	Protein Description	Fold change	*P*-value
P12242	UCP1	Mitochondrial brown fat uncoupling protein 1	22.72	0.035
Q8CGN5	PLIN1	Perilipin-1	6.06	0.019
Q05421	CYP2E1	Cytochrome P450 2E1	3.90	0.025
P05977	MYL1	Myosin light chain 1/3, skeletal muscle isoform	3.09	0.003
P97314	CSRP2	Cysteine and glycine-rich protein 2	2.81	0.019
Q08091	CNN1	Calponin-1	2.62	0.021
P13412	TNNI2	Troponin I, fast skeletal muscle	2.50	0.028
P97873	LOXL1	Lysyl oxidase homolog 1	2.36	0.043
P04919	SLC4A1	Band 3 anion transport protein	2.21	0.018
Q9QYB8	ADD2	Beta-adducin	2.07	0.008
P21956	MFGE8	Lactadherin	2.00	0.023
P02088	HBB-B1	Hemoglobin subunit beta-1	1.99	0.027
Q80X19	COL14A1	Collagen alpha-1(XIV) chain	1.96	0.039
Q9Z1T2	THBS4	Thrombospondin-4	1.96	0.024
P49222	EPB42	Protein 4.2	1.94	0.009
P15327	BPGM	Bisphosphoglycerate mutase	1.90	0.034
Q8BVA4	LMOD1	Leiomodin-1	1.90	0.019
P00920	CA2	Carbonic anhydrase 2	1.89	0.013
Q8BSM7	SLC43A1	Large neutral amino acids transporter small subunit 3	1.88	0.008
O70209	PDLIM3	PDZ and LIM domain protein 3	1.87	0.005
P58774	TPM2	Tropomyosin beta chain	1.86	0.006
P04444	HBB-BH1	Hemoglobin subunit beta-H1	1.84	0.002
O08638	MYH11	Myosin-11	1.83	0.040
Q62219	TGFB1I1	Transforming growth factor beta-1-induced transcript 1 protein	1.83	0.010
Q3UPI1	GASKBB	Golgi-associated kinase 1B	1.82	0.042
P53657	PKLR	Pyruvate kinase PKLR	1.82	0.014
P08551	NEFL	Neurofilament light polypeptide	1.82	0.024
P13634	CA1	Carbonic anhydrase 1	1.79	0.027
O09164	SOD3	Extracellular superoxide dismutase [Cu–Zn]	1.78	0.036
P28653	BGN	Biglycan	1.76	0.018
Q01149	COL1A2	Collagen alpha-2(I) chain	1.76	0.003
Q8JZU2	SLC25A1	Tricarboxylate transport protein, mitochondrial	1.75	0.035
P01942	HBA	Hemoglobin subunit alpha	1.74	0.012
Q9WV69	DMTN	Dematin	1.73	0.002
Q6PHS9	CACNA2D2	Voltage-dependent calcium channel subunit alpha-2/delta-2	1.72	0.005
O55042	SNCA	Alpha-synuclein	1.72	0.002
Q8JZW4	CPNE5	Copine-5	1.71	0.009
Q925B0	PAWR	PRKC apoptosis WT1 regulator protein	1.71	0.022
Q62000	OGN	Mimecan	1.71	0.027
Q02357	ANK1	Ankyrin-1	1.70	0.011
Q70IV5	SYNM	Synemin	1.70	0.001
Q03173	ENAH	Protein enabled homolog	1.65	0.003
Q60847	COL12A1	Collagen alpha-1(XII) chain	1.64	0.047
P08553	NEFM	Neurofilament medium polypeptide	1.64	0.014
Q4U4S6	XIRP2	Xin actin-binding repeat-containing protein 2	1.64	0.032
O88207	COL5A1	Collagen alpha-1(V) chain	1.62	0.018
Q80XB4	NRAP	Nebulin-related-anchoring protein	1.62	0.044
Q61753	PHGDH	D-3-phosphoglycerate dehydrogenase	1.61	0.002
P16110	LGALS3	Galectin-3	1.60	0.021
P18872	GNAO1	Guanine nucleotide-binding protein G(o) subunit alpha	1.60	0.008
Q7TQ62	PODN	Podocan	1.60	0.003
P13595	NCAM1	Neural cell adhesion molecule 1	1.60	0.003
Q9Z110	ALDH18A1	Delta-1-pyrroline-5-carboxylate synthase	1.57	0.003
Q99MQ4	ASPN	Asporin	1.57	0.042
P09541	MYL4	Myosin light chain 4	1.57	0.004
Q922H2	PDK3	[Pyruvate dehydrogenase (acetyl-transferring)] kinase isozyme 3, mitochondrial	1.55	0.018
Q8VHX6	FLNC	Filamin-C	1.51	0.028
Q9CYL5	GLIPR2	Golgi-associated plant pathogenesis-related protein 1	1.51	0.044
P08121	COL3A1	Collagen alpha-1(III) chain	1.50	0.023
P70290	MPP1	55 kDa erythrocyte membrane protein	1.49	0.029
Q99K41	EMILIN1	EMILIN-1	1.49	0.014
P50608	FMOD	Fibromodulin	1.49	0.050
Q62059	VCAN	Versican core protein	1.48	0.008
Q8CG19	LTBP1	Latent-transforming growth factor beta-binding protein 1	1.48	0.022
O70423	AOC3	Membrane primary amine oxidase	1.48	0.015
Q9R111	GDA	Guanine deaminase	1.48	0.001
Q61548	SNAP91	Clathrin coat assembly protein AP180	1.47	0.032
Q62523	ZYX	Zyxin	1.47	0.036
Q8BHC0	LYVE1	Lymphatic vessel endothelial hyaluronic acid receptor 1	1.47	0.013
Q91XF0	PNPO	Pyridoxine-5'-phosphate oxidase	1.46	0.010
Q9JMD3	STARD10	START domain-containing protein 10	1.45	0.040
P59644	INPP5J	Phosphatidylinositol 4,5-bisphosphate 5-phosphatase A	1.45	0.033
P61922	ABAT	4-aminobutyrate aminotransferase, mitochondrial	1.45	0.015
O70373	XIRP1	Xin actin-binding repeat-containing protein 1	1.44	0.044
P51885	LUM	Lumican	1.44	0.015
Q99JR1	SFXN1	Sideroflexin-1	1.44	0.012
Q62465	VAT1	Synaptic vesicle membrane protein VAT-1 homolog	1.42	0.040
Q9QXS6	DBN1	Drebrin	1.42	0.029
P06802	ENPP1	Ectonucleotide pyrophosphatase/phosphodiesterase family member 1	1.42	0.012
Q99L88	SNTB1	Beta-1-syntrophin	1.41	0.002
Q9D783	KLHL40	Kelch-like protein 40	1.41	0.034
Q3URD3	SLMAP	Sarcolemmal membrane-associated protein	1.40	0.026
P97465	DOK1	Docking protein 1	1.40	0.036
Q9WUX5	IRAG1	Inositol 1,4,5-triphosphate receptor associated 1	1.39	0.049
Q9WVB4	SLIT3	Slit homolog 3 protein	1.39	0.043
P11627	L1CAM	Neural cell adhesion molecule L1	1.39	0.002
P14873	MAP1B	Microtubule-associated protein 1B	1.39	0.032
Q9Z2H5	EPB41L1	Band 4.1-like protein 1	1.39	0.008
Q6P6L0	FILIP1L	Filamin A-interacting protein 1-like	1.39	0.007
P08032	SPTA1	Spectrin alpha chain, erythrocytic 1	1.38	0.021
Q921U8	SMTN	Smoothelin	1.38	0.010
P97872	FMO5	Flavin-containing monooxygenase 5	1.37	0.027
P24638	ACP2	Lysosomal acid phosphatase	1.36	0.010
P11352	GPX1	Glutathione peroxidase 1	1.36	0.007
P15508	SPTB	Spectrin beta chain, erythrocytic	1.36	0.006
P35385	HSPB7	Heat shock protein beta-7	1.36	0.003
Q9R0P5	DSTN	Destrin	1.35	0.037
Q8BHZ0	CYRIA	CYFIP-related Rac1 interactor A	1.35	0.045
Q8R242	CTBS	Di-N-acetylchitobiase	1.34	0.038
Q04857	COL6A1	Collagen alpha-1(VI) chain	1.34	0.027
Q9DBJ1	PGAM1	Phosphoglycerate mutase 1	1.34	0.016
Q9DCS2	METTL26	Methyltransferase-like 26	1.34	0.011
Q9CQL1	MAGOHB	Protein mago nashi homolog 2	1.34	0.023
Q62148	ALDH1A2	Retinal dehydrogenase 2	1.33	0.037
P46412	GPX3	Glutathione peroxidase 3	1.33	0.000
Q9DBE0	CSAD	Cysteine sulfinic acid decarboxylase	1.33	0.046
P84309	ADCY5	Adenylate cyclase type 5	1.33	0.030
Q9JKB3	YBX3	Y-box-binding protein 3	1.33	0.029
Q9DBU3	RIOK3	Serine/threonine-protein kinase RIO3	1.32	0.027
P81117	NUCB2	Nucleobindin-2	1.32	0.008
Q9EQF6	DPYSL5	Dihydropyrimidinase-related protein 5	1.32	0.001
Q8K2A7	INTS10	Integrator complex subunit 10	1.31	0.025
P23953	CES1C	Carboxylesterase 1C	1.31	0.023
Q68FD5	CLTC	Clathrin heavy chain 1	1.31	0.035
P22437	PTGS1	Prostaglandin G/H synthase 1	1.31	0.017
Q9CZS1	ALDH1B1	Aldehyde dehydrogenase X, mitochondrial	1.31	0.049
Q8C1D8	IWS1	Protein IWS1 homolog	1.30	0.027
P32261	SERPINC1	Antithrombin-III	1.30	0.045

### Pathway analysis of differentially expressed proteins in hearts of DEP-exposed *ApoE*^−/−^ mice

**Figure 3:**
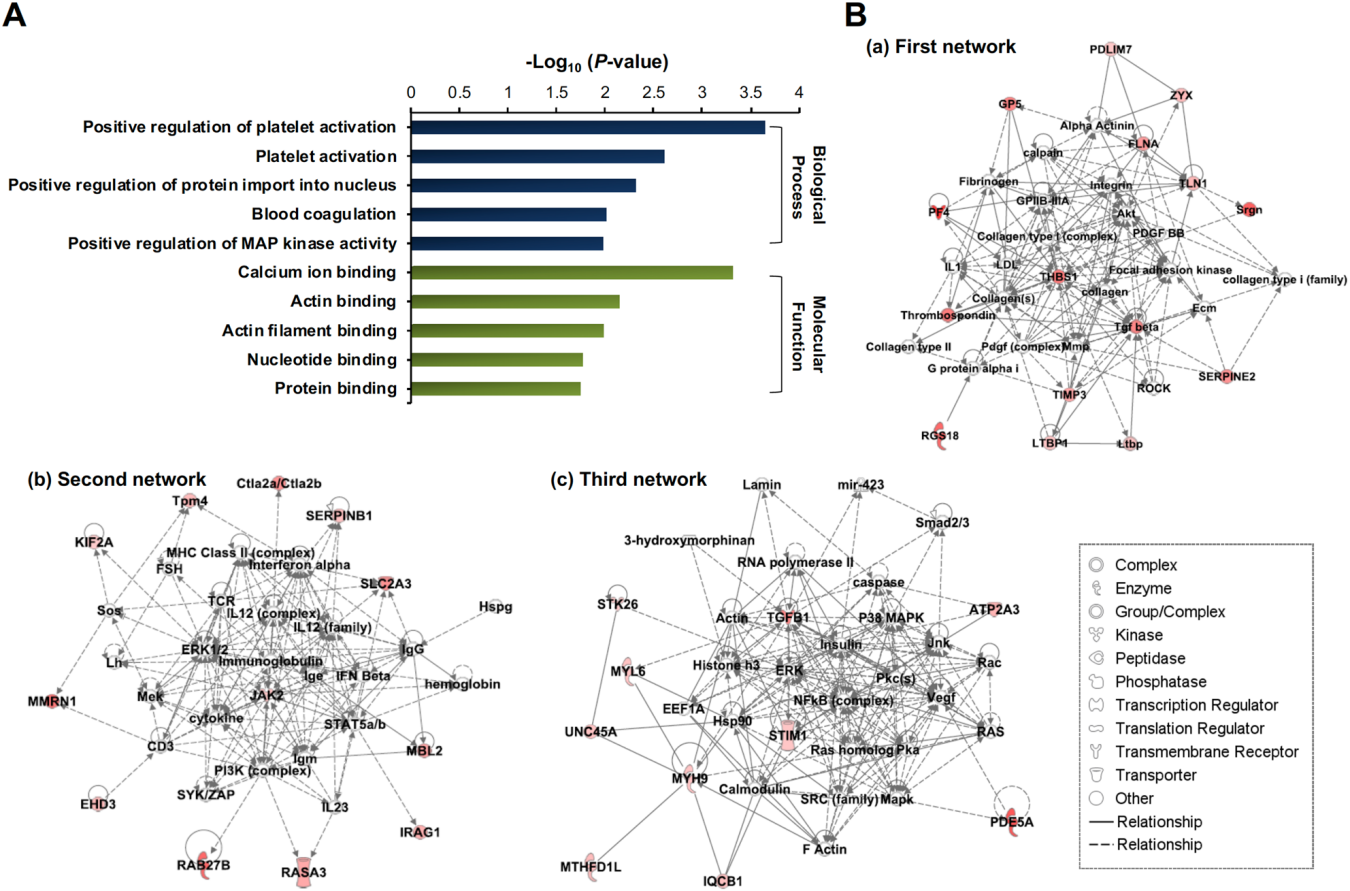
Bioinformatic analysis of differentially expressed proteins. (**A**) Top five biological process and molecular function terms enriched in the 39 proteins significantly increased (*P*-value < 0.05, > 1.3-fold) in DEP-treated *ApoE*^*−/−*^ mice relative to PBS-treated *ApoE*^*−/−*^ mice, as identified by Gene Enrichment (GO) enrichment analysis. (**B**) Top three interaction networks generated by ingenuity pathway analysis (IPA) are shown, respectively, in (a), (b), and (c). Proteins that significantly increased (*P*-value < 0.05, > 1.3-fold) in DEP-treated *ApoE*^*−/−*^ mice in comparison to PBS-treated *ApoE*^*−/−*^ mice are represented in red. TGF beta, ERK1/2, and TGFB1/NFκB were identified as the major hubs in each network. Straight lines denote direct interactions, and dashed lines denote indirect interactions.

GO enrichment analysis of the 39 proteins upregulated (> 1.3-fold, *P*-value < 0.05) in DEP-treated *ApoE*^*−/−*^ mice in comparison to PBS-treated *ApoE*^*−/−*^ mice revealed that positive regulation of platelet activation and platelet activation terms were enriched in the top five biological process terms, while calcium ion binding and actin binding were enriched in the top five molecular function terms (Fig. 3A). The 39 proteins significantly increased in DEP-treated *ApoE*^*−/−*^ mice were subjected to IPA analysis (Table 3), revealing that the top five molecular and cellular functions in order of significance were ‘cell-to-cell signaling and interaction,’ ‘cellular function and maintenance,’ ‘cellular development,’ ‘cellular growth and proliferation,’ and ‘cell morphology.’ The two diseases and disorders most related were ‘inflammatory response’ and ‘cardiovascular disease,’ indicating that DEP exposure could cause CVDs in *ApoE*^*−/−*^ mice. Further, ‘cardiac arteriopathy,’ ‘cardiac dilation,’ ‘cardiac enlargement,’ ‘congenital heart anomaly,’ and ‘cardiac inflammation’ were categorized in the cardiotoxicity functions analyzed in the IPA program (Table 3). The top three networks from IPA analysis results were TGFβ (12 proteins), extracellular signal-regulated kinase (ERK)1/2 (12 proteins), and transforming growth factor beta 1 (TGFB1)/NFκB (10 proteins), identifying these signaling pathways as the major hubs of DEP-induced heart damage (Fig. 3B).

**Table 3:** Ingenuity pathway analysis of proteins upregulated by DEP exposure in hearts of *ApoE*^*−/−*^ mice.

Molecular and cellular functions	*P*-value^†^	No.^‡^
Cell-to-cell signaling and interaction	1.69E−03–1.58E−13	19
Cellular function and maintenance	1.66E−03–1.55E−07	19
Cellular development	1.66E−03–2.52E−07	21
Cellular growth and proliferation	1.66E−03–2.52E−07	22
Cell morphology	1.66E−03–5.65E−07	17

Diseases and disorders		
Inflammatory response	1.66E−03–5.29E−13	26
Cardiovascular disease	1.68E−03–1.53E−12	18
Hematological disease	1.66E−03–1.53E-12	14
Organismal injury and abnormalities	1.68E−03–1.53E−12	36
Renal and urological disease	1.66E−03–3.66E−07	13

Cardiotoxicity functions		
Cardiac arteriopathy	2.46E−02–8.31E−05	6
Cardiac dilation	1.27E−01–1.47E−04	6
Cardiac enlargement	2.66E−01–1.47E−04	6
Congenital heart anomaly	5.17E−02–2.77E−04	4
Cardiac inflammation	9.64E−02–1.66E−03	1

Associated network functions	Score*(No.^‡^)	
Hematological system development and function, cell-to-cell signaling and interaction, inflammatory response	28 (12)	
Cell morphology, cellular assembly and organization, cellular function and maintenance	28 (12)	
Cardiovascular disease, hematological disease, organismal injury and abnormalities	22 (10)	

^†^*P*-value is displayed in E notation: aEb indicates a value of a × 10^b^.

^‡^Number of molecules involved.

*Scores were derived from *P*-value and indicate the likelihood of the mapped genes in a network being identified together due to random chance (score = −log_10_*P*).

### Validation of proteomics findings by western blotting

**Figure 4:**
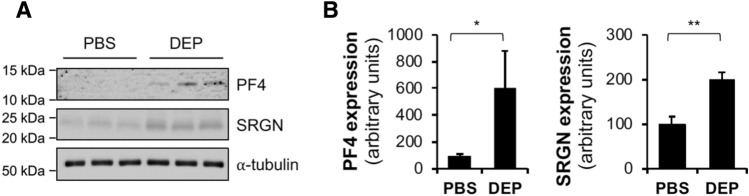
Confirmation of differentially expressed proteins by western blotting. (**A**) Expression of PF4 and SRGN in hearts of *ApoE*^*−/−*^ mice exposed to PBS or DEP. Membranes were cut prior to hybridization with antibodies. While the cropped images are seen, uncropped blots are provided in Supplementary Information. (**B**) Quantification of PF4 and SRGN protein levels normalized to ⍺-tubulin. *P < 0.05, **P < 0.02, two-tailed Student’s *t* test.

To validate the proteomic data, we performed western blotting for two mechanistically relevant upregulated proteins, PF4 (Q9Z126) and SRGN (P13609). These proteins were selected due to their known regulatory roles in CVDs-related platelet activation^22–26^. Upregulation of PF4 (Q9Z126) and SRGN (P13609) protein levels was confirmed by western blotting, with comparable relative expression patterns to those of proteomic analysis (Fig. 4A and B). These data validated important mechanistic findings initially identified by proteomics analysis, and also supported the reliability of the proteomics data set.

## Discussion

Previous epidemiological studies identified that environmental PM levels positively correlate with the severity and mortality rates of CVDs^27,28^. Specifically, several recent studies have demonstrated that PM exposure causes heart dysfunction by inducing cardiomyocyte apoptosis and production of inflammatory cytokines^29–31^ but the regulatory mechanisms for these pathologies remain incompletely understood. To delineate the regulatory mechanisms of PM-induced heart dysfunction, it is necessary to identify candidate proteins potentially responsible for development and progression of heart diseases. However, most studies investigating PM-related human disease have focused on differentially expressed proteins in lung, skin, and brain tissues^32–34^ and heart metabolite levels^35^. To further evaluate these pathologies, we established mouse models by exposing WT mice and *ApoE*^*−/−*^ mice to DEP, with AngII-infused *ApoE*^*−/−*^ mice as a positive control for robust heart fibrosis. While 100 μg of DEP was administered intratracheally into mice every 3 days for 25 days for induction of heart fibrosis, we had to select a specific dose of DEP because there were no established standard protocols to study the adverse effects of DEP in vivo. Previous studies used different protocols to investigate the adverse effects of DEP in vivo. Lee et al. exposed 50 µg of DEP to C57BL/6 mice by intratracheal instillation twice a week for 4 weeks and found that this experimental protocol caused the DNA damage in lung^36^. Another study showed that intratracheal instillation of 100 µg DEP on day 1 and day 4 and DEP + lipopolysaccharide (20 µg) on day 7 induced lung inflammation in a mouse model^37^. We were interested in investigating effects on mouse heart under the exposure of DEP. Thus, we hypothesized that more DEP had to be used because some portions of DEP injected into tracheal route would be trapped in airway or lung and the remaining portions of DEP would penetrate the respiratory system, circulate in blood vessels, and cause dysfunctions in heart. Although there was a previous study in which 200 µg of DEP was intratracheally instilled into C57BL/6 mice^38^, we selected 100 µg of extracted DEP in a single injection and repeated eight total times to induce potential heart damage. Heart protein expression analysis using LC–MS/MS was used to identify candidate proteins related to heart dysfunction in these models. Specifically, to identify differentially expressed proteins affected by DEP exposure in the *ApoE*^*−/−*^ mouse model of CVDs, we used TMT as an isobaric labeling mass tags that allowed simultaneous quantification of multiple samples and is more precise than label-free quantification^39,40^. This approach was used for quantitative analysis in combination with high-resolution mass spectrometry.

Multiple delivery approaches have been used to expose animal models to PM, including inhalation, intratracheal instillation, and nasal inoculation. Inhalation is the most physiologically relevant method that can assess the real effects of human exposure to ambient PM but requires significant quantities of PM and a costly exposure chamber^41^. Contrastingly, the cost of intratracheal instillation is relatively low, and the components of intratracheally administered PM translocate to extrapulmonary tissue from the bloodstream and cause DNA damage in tissue^42,43^. Thus, many studies use intratracheal instillation to investigate the effect of PM on tissue damage, including the effects of PM on the cardiovascular system^8,9,31,44^. Furthermore, the intratracheal delivery approach also delivers particles to the lung more efficiently than the intranasal delivery approach^45,46^. Based on these studies, we utilized intratracheal DEP instillation even if the intratracheal instillation is not the most appropriate delivery of PM to study the effects of human exposure to ambient PM. Originally, we investigated differentially expressed proteins in hearts of WT mice exposed to DEP. However, we identified only 17 differentially expressed proteins meeting the criteria of |fold change|> 1.3 and *P*-value < 0.05 (Table S5). Myosin-3 (P13541, MYH3, > 1.43-fold) was increased in WT mice exposed to DEP. When mutated in the tail domain, MYH3 contributes to atrial septal defects^47^, but as a whole, the 17 differentially expressed proteins were insufficient for bioinformatic pathway analysis. Therefore, we performed bioinformatic analysis by exposing *ApoE*^*−/−*^ mice, which are more susceptible to CVDs^14,44^, to DEP and conducting bioinformatic pathway analysis. Bioinformatic analysis of heart tissue from PBS- and DEP-treated *ApoE*^*−/−*^ mice identified 52 differentially expressed proteins, with 39 proteins upregulated and 13 proteins downregulated by DEP treatment (Fig. 2). Among the upregulated proteins, five proteins, including latent-transforming growth factor beta-binding protein 1 (Q8CG19), were also upregulated in the well-known heart fibrosis mouse model of AngII infusion into *ApoE*^*−/−*^ mice.

Among the 39 proteins upregulated in DEP-treated *ApoE*^*−/−*^ mice (Table 1), platelet factor 4 (PF4, Q9Z126) was most highly upregulated (> 2.94-fold increase). PF4, also known as chemokine (C-X-C motif) ligand 4 (CXCL4), is an abundant platelet alpha-granule CXC chemokine released during platelet activation^22^. PF4 binds and neutralizes the glycosaminoglycan heparan sulfate, promoting platelet aggregation and thrombus formation^22^. Platelet activation at vessel injury sites prevents excessive bleeding and regulates hemostasis^48^. However, platelet hyperactivation has pathological roles in multiple human diseases, including CVDs such as myocardial infarction and atherosclerosis. For example, platelet function is elevated in patients with ST-segment elevation acute myocardial infarction (STEMI)^49^. PF4 released from platelets binds the LDLR, preventing LDL endocytosis and increasing vessel LDL retention time, which potentially increases LDL oxidation (ox-LDL)^23^. Monocyte uptake of ox-LDL transforms monocytes into foam cells and contributes to early atherosclerosis onset^50^. Furthermore, PF4 increases expression of E-selectin, which regulates endothelial inflammation and atherosclerosis^24,51^. This suggests that increased PF4 expression reflects platelet activation and ultimately leads to heart and blood vessel disease. While PDE5A (Q8CG03) was found to be highly upregulated by DEP exposure in *ApoE*^*−/−*^ mice (> 2.41-fold increase), inhibition of PDE5A was previously found to prevent cardiac fibrosis by regulating the Smad signaling cascade^52^. It was also shown that PDE5A is involved in bleomycin-induced pulmonary fibrosis^53^, suggesting that upregulation of PDE5A by DEP exposure in *ApoE*^*−/−*^ mice could be associated with tissue fibrosis. In addition, *PDE5A*^*−/−*^ mice showed reduced cardiac rupture and inflammatory response after myocardial infarction^54^, and showed prolonged tail bleeding time and delayed thrombus formation, indicating that PDE5A regulates the function of platelet^55^. We identified that serglycin (SRGN, P13609) was highly upregulated by DEP exposure in *ApoE*^*−/−*^ mice (> 2.30-fold increase). SRGN is an intracellular proteoglycan that colocalizes with PF4 in platelet ⍺-granules^25,56^. PF4 levels are profoundly decreased in platelets from *Srgn*^*−/−*^ mice, and platelet aggregation potential is inhibited^26^, suggesting that SRGN upregulation could contribute to platelet activation. DEP exposure upregulated bridging integrator 2 (BIN2, D3Z6Q9) (> 2.10-fold increase). BIN2 interacts with stromal interaction molecule 1 and inositol trisphosphate receptor on platelets and regulates Ca^2+^ signaling^57^. Deletion of platelet BIN2 impairs thrombus formation, suggesting that BIN2 contributes to platelet activation^57^. We further identified that DEP exposure upregulated multimerin-1 (MMRN1, B2RPV6) and platelet glycoprotein V (GP5, O08742) (MMRN1 > 2.02-fold increase, GP5 > 1.87-fold increase). MMRN1 is a member of the EMILIN/multimerin family and is present in ⍺-granules of resting platelets and secretary granules of endothelial cells^58,59^. Following platelet activation, MMRN1 is secreted and binds fibrillar collagen to support platelet adhesion and thrombus formation^60–62^. GP5 is an abundant glycoprotein at the platelet surface, and is reported to bind collagen and mediate platelet adhesion and aggregation^63^. Further, soluble GP5 is a potential marker of thrombosis in ischemic stroke^64^. These prior studies suggest that DEP upregulation of MMRN1 and GP5 could activate platelets in *ApoE*^*−/−*^ mice. Taken together, the prior and present findings suggest potential platelet activation in hearts of DEP-exposed *ApoE*^*−/−*^ mice, which is consistent with GO enrichment analysis findings (Fig. 3A).

Mild fibrosis was present in hearts of *ApoE*^*−/−*^ mice exposed to DEP (Fig. 1B, C). Consistent with this observation, transforming growth factor beta-1 proprotein (TGFB1 or TGFβ1, P04202) and latent-transforming growth factor beta-binding protein 1 (LTBP1, Q8CG19) were also upregulated in hearts of *ApoE*^*−/−*^ mice exposed to DEP (TGFB1 > 1.88-fold increase, LTBP1 > 1.36-fold increase). IPA findings identified that TGFβ and TGFB1 were major hubs for DEP-induced heart fibrosis, and were in the first and third interaction networks (Fig. 3B). Cardiac fibrosis is characterized by transformation of cardiac fibroblasts into ⍺SMA-expressing myofibroblasts and subsequent dysregulation of extracellular matrix (ECM) protein expression, promoting cardiac dysfunction^65^. TGFβ, which is synthesized as a proprotein and processed into a mature form^66^, plays a critical role in progression of cardiac disease^67^. TGFβ induces transformation of fibroblasts to myofibroblasts and increases ECM protein expression^68^. TGFβ causes cardiac dysfunction by inducing cardiomyocyte hypertrophy and the endothelial-to-mesenchymal transition (EndMT)^69–71^. Further, age-associated myocardial fibrosis is decreased in TGFβ1-deficient mice, and ventricular fibrosis is exacerbated in TGFβ1-overexpressing mice^72,73^. Heart levels of LTBP1 were upregulated by both DEP exposure and AngII infusion (DEP exposure > 1.36-fold and AngII infusion > 1.48-fold increases of LTBP1 expression). LTBP1 forms a complex with TGFβ and targets extracellular fibrillin and fibronectin, where TGFβ is sequestered in a latent state^74–76^. When this complex is degraded by proteases or other stimuli, latent TGFβ is released and activates neighboring cells by binding its receptor, inducing multiple TGFβ-activated cellular phenotypes, including fibrosis^76^. The findings of the present study support potential involvement of TGFβ in DEP-induced heart fibrosis of *ApoE*^*−/−*^ mice.

While the proteins including PF4, PDE5A, MMRN1, and GP5 that showed statistically significant increases in hearts of DEP-exposed *ApoE*^*−/−*^ mice were known to be associated with thrombosis, thrombosis is a blood clot within blood vessel that disrupts the blood flow^77^. Although the roles of platelet in thrombosis, fibrosis, and blood vessel remodeling were reported^9,78,79^, the direct evidence for the relationship between thrombosis and fibrosis seems to be limited. Disturbed blood flow pattern induces vascular inflammation and EndMT^80,81^. Zeisberg et al. reported that cardiac fibrosis is associated with the appearance of fibroblast originated from endothelial cells^82^. It was shown that inhibition of EndMT by administration of bone morphogenic protein 7 attenuated the progression of cardiac fibrosis^82^. This result implies that EndMT induced by thrombosis-mediated blood flow disruption may be able to contribute to the cardiac fibrosis. However, more studies are required to elucidate the relationship between thrombosis and cardiac fibrosis.

We previously characterized the chemical components of DEP^13^. DEP consist of organic carbon, ions, elemental carbon, and elements. Because we extracted filter-collected particles with PBS in the present study, intratracheally instilled DEP would be likely to contain water soluble fractions such as ions and some elements. However, the adverse effects of individual ions and elements on the heart are not clear. Although phosphorus is a candidate element for cardiac fibrosis^83^, further studies are required to examine the effects of each component on heart fibrosis and platelet activation.

Since previous studies showed that PM exposure to heart in mouse model induced cardiac fibrosis through TGFβ and Smad3 signaling cascade^84,85^ and that PM exposure could induce platelet activation and thrombosis^9,11^, our findings in the current study may have some similarity to the previous reports. However, we think that our proteomics-based approach allows for discovery of new protein targets such as PF4 and PED5A using proteomic analysis because most of the previous studies focused on exploring some known targets that were related to cardiac fibrosis and platelet activation/thrombosis.

In conclusion, we identified that candidate proteins and biomechanical pathways which could contribute to heart damage were induced by DEP exposure in the *ApoE*^*−/−*^ mouse model. Specifically, we focused on platelet-dependent pathways in heart dysfunction because prior studies have suggested that air pollution has prothrombotic effects that contribute to human diseases^10,86^. Thus, future studies will be aimed at determining the precise roles of proteins that regulate platelet activation and thrombosis such as PF4, SRGN, BIN2, MMRN1, and GP5 in DEP-induced heart damage.

## Methods

### PM preparation

Engine exhaust particles were produced with a diesel engine (498 cc, DG8500SE, Hi-Earns Mechanical and Electrical Co., Ltd., Changzhou, China) and collected on filters using a low-volume PM2.5 sampler (URG-2000-30EH, URG, Chapel Hill, NC, USA) at a flow rate of 16.7 L/min for 30 min as described previously^13^. The mass of the collected PM2.5 was determined based on the weight of the filter, which was equilibrated at 21 ± 2 °C and relative humidity 35 ± 5% for 24 h before and after collection. The weights of the filter that were measured before and after DEP collection were 85.1200 mg and 194. 3967 mg, respectively. DEP with diameter < 2.5 μm were collected on a glass fiber filter (Pall Corporation, Port Washington, NY, USA) and extracted with phosphate-buffered saline (PBS, Sigma-Aldrich, St. Louis, MO, USA). Extracted DEP were filtered through a 0.2 μm PTFE syringe filter (Sartorius AG, Germany) prior to treating mice.

### Animal experiments

Animal experiments were performed by Knotus (Incheon, Republic of Korea) and approved by the Institutional Animal Care and Use Committee (KNOTUS 20-KE-017). All methods for the animal experiments were performed in accordance with the relevant guidelines and regulations. Animals were euthanized by isoflurane and all efforts were taken to minimize their suffering. These experimental procedures are consistent with those outlined in the ARRIVE guidelines. Seven-week-old male apolipoprotein E knockout (*ApoE*^*−/−*^) mice or C57BL/6 wild-type (WT) mice were purchased from Jackson Laboratory (Bar Harbor, ME, USA) and adapted to the facility for 1 week. Mice were divided into five groups (n = 3 mice/group): (1) intratracheal administration of PBS in WT mice (PBS-treated WT mice), (2) intratracheal administration of DEP in WT mice (DEP-treated WT mice), (3) intratracheal administration of PBS and saline infusion in *ApoE*^*−/−*^ mice (PBS-treated *ApoE*^*−/−*^ mice), (4) intratracheal administration of DEP and saline infusion in *ApoE*^*−/−*^ mice (DEP-treated *ApoE*^*−/−*^ mice), and (5) intratracheal administration of PBS and angiotensin II infusion (AngII, Sigma-Aldrich) in *ApoE*^*−/−*^ mice (AngII-infused *ApoE*^*−/−*^ mice). For infusion, mice were anesthetized, and an osmotic pump (Alzet, Cupertino, CA, USA) filled with AngII (1000 ng/kg/min) or saline was subcutaneously administered on Day 0 such that AngII or saline was perfused throughout the experiment. From the following day (Day 1), mice were anesthetized and treated with extracted DEP (100 µg) that included soluble components in PBS and particles smaller than 0.2 µm or PBS via intratracheal administration. Mice were treated with DEP or PBS every 3 day for 25 days (eight total exposures); 3 days following the final treatment, they were euthanized. Hearts were removed, fixed in 10% neutral buffered formalin, and embedded in paraffin. Tissue sections (5 μm) were cut, deparaffinized, and subjected to Masson’s trichrome staining. The fibrotic area was observed using an EVOS M5000 Imaging system (Invitrogen, Carlsbad, CA, USA), and the fibrotic area was quantified using Image J software. Briefly, the blue positive area was divided by the total area for each field, and the average value, which means the fibrotic area, was calculated for each group^84^. Analysis was performed by blinded persons to the groups. When hearts were removed, some part of hearts were frozen and used for the proteomic analysis or western blotting.

### In-solution digestion for proteomic analysis

Heart tissues were first lysed in lysis buffer (7 M Urea, 2 M Thiourea, 1 mM EDTA, 150 mM NaCl, 50 mM Tris–HCl pH 7.5, and protease inhibitor cocktail (Roche Diagnostics, Mannheim, Germany)) using a probe-type sonicator (Sonics & Materials, Newtown, CT, USA). Protein concentrations were determined using a Bradford protein assay (Bio-Rad Laboratories, Inc. Hercules, CA, USA). Prior to in-solution digestion, all samples were diluted using 25 mM ammonium bicarbonate (ABC) to equalize concentrations. Urea was added to samples to a final concentration of 8 M. Samples were then reduced with 5 mM tris(2-carboxyethyl) phosphine hydrochloride and alkylated with 10 mM iodoacetamide. After samples were treated with 25 mM ABC to decrease the urea concentration to < 1 M, samples were digested with lysyl endopeptidaseR (Lys-C, Fujifilm Wako Pure Chemical Corporation, Osaka, Japan) at an enzyme/substrate ratio of 1 mAU Lys-C per 50 μg total protein at 30 °C for 2 h^87^. Trypsin was then added to the samples (Promega, Madison, WI, USA) at a protease to substrate ratio of 1:50 (wt/wt) and incubated at 37 °C overnight. Digested peptide samples were desalted using a Sep-Pak tC18 cartridge (Waters Corporation, Milford, MA, USA) and subsequently dried in a miVAC vacuum concentrator (Genevac Ltd., Ipswich, UK). After samples were resuspended in 100 mM triethyl ammonium bicarbonate (TEAB), peptide concentrations were determined using a quantitative colorimetric peptide assay kit (Thermo Fisher Scientific, Rockford, IL, USA).

### Tandem mass tag (TMT) labeling and basic pH reversed‑phase liquid chromatography

After TMTpro 16plex label reagents (0.5 mg per vial, Thermo Fisher Scientific, Rockford, IL, USA, VF304377 (lot number)) were resuspended in 42 μL of anhydrous acetonitrile (ACN), 20 μL of each label reagent was added to 30 μg of each peptide samples for labeling and the labeling reactions were made for 60 min at room temperature. Then, 5 μL of 5% hydroxylamine in 100 mM TEAB was added to each peptide sample and incubated for 15 min to quench the labeling reactions. Equal amounts of TMT-labeled samples were then combined, desalted using a Sep-Pak tC18 cartridge, and dried in a miVAC vacuum concentrator. TMT-labeled peptide samples were resuspended in 10 mM ammonium formate (AF), and peptide concentrations were determined using a quantitative colorimetric peptide assay kit. Then, 410 μg peptide sample was loaded onto an X-Bridge peptide BEH C18 column (4.6 mm i.d. × 250 mm length; pore size 130 Å; particle size 3.5 μm, Waters Corporation) and fractionated by basic pH reversed-phase liquid chromatography using an Agilent 1290 Infinity liquid chromatography (LC) system (Agilent Technology, Santa Clara, CA). Peptides were separated at a flow rate of 0.5 mL/min with the following gradient conditions: 0 min 100% buffer A (10 mM AF, pH 10) and 0% buffer B (10 mM AF, pH 10 in 90% acetonitrile), 0–10 min 0–5% B, 10–48.5 min 5–40% B, 48.5–62.5 min 40–70% B, 62.5–72.5 min 70% B, 72.5–82.5 min 70–5% B, and 82.5–92.5 min 5% B. Fractionation was conducted by collecting 96 wells (1 well/0.8 min, Restek corporation, Bellefonte, PA, USA) during the chromatographic run (10–82.5 min). The resultant 96 fractions were pooled to 24 concatenated fractions, dried, and subsequently resuspended in 17.08 μL 0.4% acetic acid.

### Liquid chromatography and tandem mass spectrometry (LC–MS/MS) analysis

Three µg fractionated peptide samples were injected into a trap column (2 cm × 75 µm i.d., 100 Å, 3 µm) and separated on a reversed-phase Acclaim PepMap RSLC C18 column (50 cm × 75 µm i.d., 100 Å, 2 µm) using an UltiMate 3000 RSLCnano System (Thermo Fisher Scientific). Column temperature was constantly set to 50℃ with a column heater The operating flow rate was 300 nL/min with the following gradient conditions: 0 min 95% buffer A (100% water with 0.1% formic acid) and 5% buffer B (100% acetonitrile with 0.1% formic acid), 0–4 min 5% B, 4-13 min 5–10% B, 13–150 min 10–25% B, 150–155 min 25–28% B, 155–160 min 28–40% B, 160–165 min 40–80% B, 165–170 min 80%B, 170–170.1 min 80–5% B, and 170.1–180 min 5–0%B. The nano UHPLC system was coupled to an Orbitrap Eclipse Tribrid Mass Spectrometer (Thermo Fisher Scientific). MS1 data were collected using an Orbitrap (120,000 resolution; scan range 350–2000 m/z; maximum injection time 50 ms; automatic gain control (AGC) 4 × 10^5^). Determined charge states between 2 and 6 were required for sequencing, and a 30 s dynamic exclusion window was used. Data-dependent ‘top 20’ MS2 scans were performed in an 0.5 Da ion trap isolation window with collision-induced dissociation (CID) fragmentation (NCE 35%; maximum injection time 35 ms; AGC 1 × 10^4^). MS3 quantification scans were performed using the multinotch MS3-based TMT method^88^ (ten synchronous precursor selection (SPS) ions; 50,000 resolution; NCE 55% for higher-energy collisional dissociation (HCD); maximum injection time of 130 ms; AGC 1.5 × 10^5^).

### Data processing for protein identification and quantification

MS raw files were searched against the SwissProt mouse database (November 2020) with 17,196 entries using Proteome Discoverer software (version 2.4, Thermo Fisher Scientific). The search criteria were set to a mass tolerance of 10 ppm for MS data and 0.6 Da for MS/MS data with fixed modifications for cysteine carbamidomethylation (+ 57.021 Da), TMT on lysine residues and peptide N termini (+ 304.207 Da), and variable modification of methionine oxidation (+ 15.995 Da). The false discovery rate (FDR) was set to 0.01 for identification of peptides and proteins. All proteins were identified by one or more unique peptides. Reporter ion quantification was performed with a 20 ppm mass tolerance, and signal-to-noise ratio values of reporter ions were used for peptide quantification. Only spectra with an average reporter signal-to-noise ratio threshold ≥ 10 across 16 TMTpro 16-plex channels were considered for quantification. The signal-to-noise ratio values of each reporter ion channel were summed across all quantified proteins and normalized to make the summed signal-to-noise ratio values of each channel equal across all 16 channels. The normalized signal-to-noise ratio values were first log-transformed, and missing values were then replaced using values computed from the normal distribution with a width of 0.3 and a downshift of 1.8. Proteins exhibiting statistical significances between two types of mouse groups (Student’s *t* test comparison of the log_2_(normalized signal-to-noise ratio) values *P* < 0.05) were identified. Statistical significance was calculated using Perseus software (1.6.14.0)^89^.

### Bioinformatics analysis

A volcano plot of peptides with quantitative information was constructed using Perseus (1.6.14.0) according to statistical *P*-value (-log_10_*P*-value as y-axis) and relative abundance ratios (log_2_fold change as x-axis) between PBS- and DEP-treated *ApoE*^*−/−*^ mice. For hierarchical clustering of proteins with statistically significant changes (*P*-value < 0.05, > 1.3-fold) between PBS- and DEP-treated *ApoE*^*−/−*^ mice, log_2_(normalized signal-to-noise ratio) values were first normalized using the z-score, and subsequent clustering of both columns and rows was conducted based on Euclidean distance with the average linkage method using Perseus (1.6.14.0). GO functional classifications of proteins with statistically significant upregulation (*P*-value < 0.05, > 1.3-fold) between PBS- and DEP-treated *ApoE*^*−/−*^ mice were analyzed using DAVID software (http://david.abcc.ncifcrf.gov) to identify GO terms that were significantly enriched in the proteins. Additionally, IPA software (data version 81348237; QIAGEN, Redwood City, CA) was used to analyze molecular and cellular functions and the associated network functions of proteins exhibiting statistically significant increases in DEP-treated *ApoE*^*−/−*^ mice relative to PBS-treated *ApoE*^*−/−*^ mice.

### Western blotting

Frozen heart tissue was lysed in cell lysis buffer (Cell Signaling Technology, Beverly, MA, USA) supplemented with protease inhibitor cocktail (Sigma-Aldrich). Equal amounts of cell lysates were separated by sodium dodecyl sulfate–polyacrylamide gel electrophoresis. Proteins were transferred onto Immuno-Blot polyvinylidene difluoride membranes (Bio-Rad Laboratories) and subsequently blocked in 5% nonfat milk (Santa Cruz Biotechnology, Santa Cruz, CA, USA) in 0.1% Tween 20-containing tris-buffered saline (TBS) for 1 h. Membranes were cut to include the corresponding protein molecular weight sizes and incubated overnight at 4 °C with the appropriate primary antibodies. After washing three times with 0.1% Tween 20-containing TBS, membranes were incubated with horseradish peroxidase-conjugated secondary antibody (1:5,000 dilution, Santa Cruz Biotechnology) for 1 h. After washing with 0.1% Tween 20-containing TBS, signals were visualized using an ImageQuant LAS4000 mini system (GE Healthcare, Chicago, IL, USA) using Western Blotting Luminol Reagent (Santa Cruz Biotechnology). Densitometric analysis was performed using Image J software. ⍺-smooth muscle actin (SMA) antibody was purchased from Abcam (Cambridge, UK). PF4 antibody was purchased from R&D systems (Minneapolis, MN, USA). Serglycin (SRGN) antibody was purchased from Santa Cruz Biotechnology. ⍺-tubulin antibody was purchased from Sigma-Aldrich.

### Statistics

Western blotting results are presented as means ± SD. Statistical significance between two groups was evaluated using a two-tailed Student’s t test. Statistical significance between more than two groups was evaluated using one-way ANOVA. *P*-value < 0.05 was considered significant.

### Supplementary Information

Supplementary Information 1.

Supplementary Information 2.

## Data Availability

The data analyzed in the current study are available from the corresponding author on reasonable request. The mass spectrometry data have been deposited to the ProteomeXchange Consortium via the PRIDE^90^ partner repository with the dataset identifier PXD045029.
