# Supplementary material for: Proteomic analysis reveals activation of platelet- and fibrosis-related pathways in hearts of ApoE−/− mice exposed to diesel exhaust particles
Source: Sci Rep. 2023 Dec 19;13:22636. doi: 10.1038/s41598-023-49790-y (PMC10730529; doi:10.1038/s41598-023-49790-y)
Supplement: Supplementary file 1 — Supplementary Information 1. [file 41598_2023_49790_MOESM1_ESM.pdf]

Supplementary figures

Table of contents:

Supplementary Figure S1. DEP exposure did not induce heart fibrosis in WT mice.

Supplementary Figure S2. Venn diagrams showing the numbers of proteins identified by LC-MS/MS analysis from PBS-treated WT mice, DEP-treated WT mice, PBS-treated *ApoE*$^{-/-}$ mice, DEP-treated *ApoE*$^{-/-}$ mice, and AngII-infused *ApoE*$^{-/-}$ mice.

Uncropped images of western blot results from Figure 1D and Figure 4A.

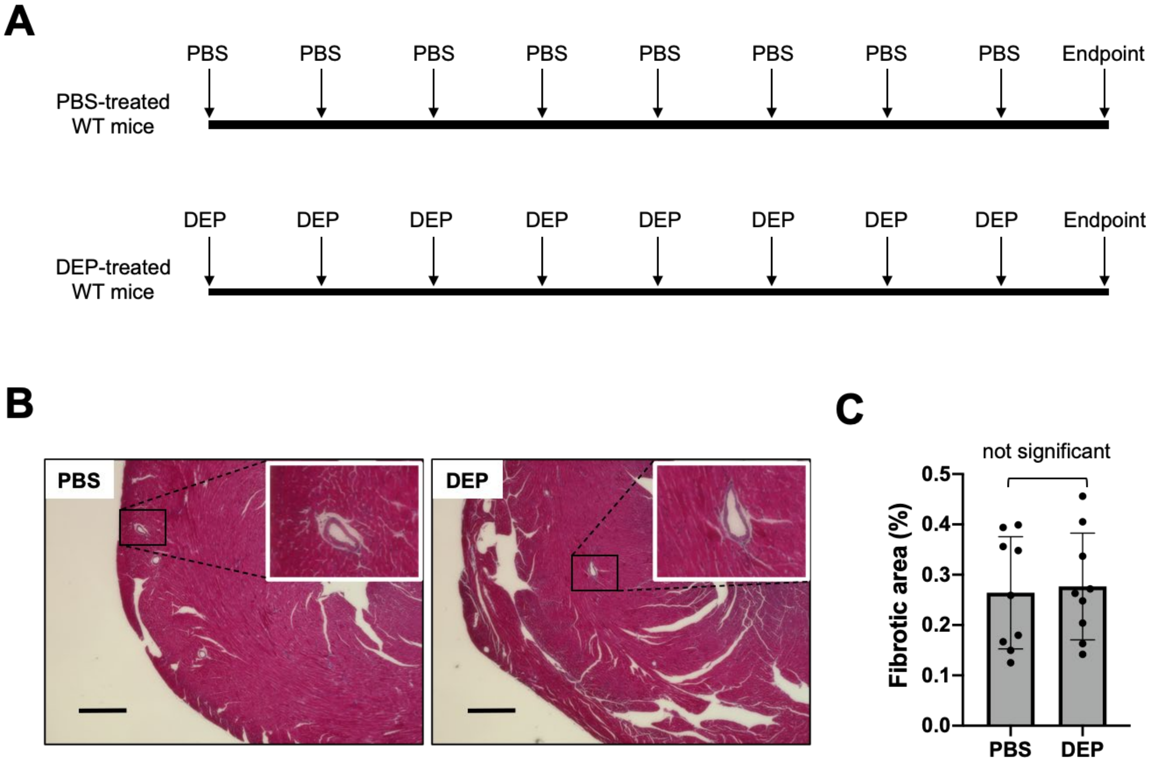

Figure S1. DEP exposure did not induce heart fibrosis in WT mice. (A) Schematic illustration of the DEP exposure protocol. PBS or DEP (100 μg) were administered via intratracheal injection eight total times at 3 day intervals. Three days following the final injection, mice were euthanized, and hearts were harvested. **(B)** Representative images of Masson's trichrome staining from hearts of PBS-treated and DEP-treated WT mice. Scale bar, 400 μm. **(C)** Fibrotic areas of each image were quantified using Image J software (n = 9). Data are presented as means ± SD. Statistical analysis was performed using a two-tailed Student's t test.

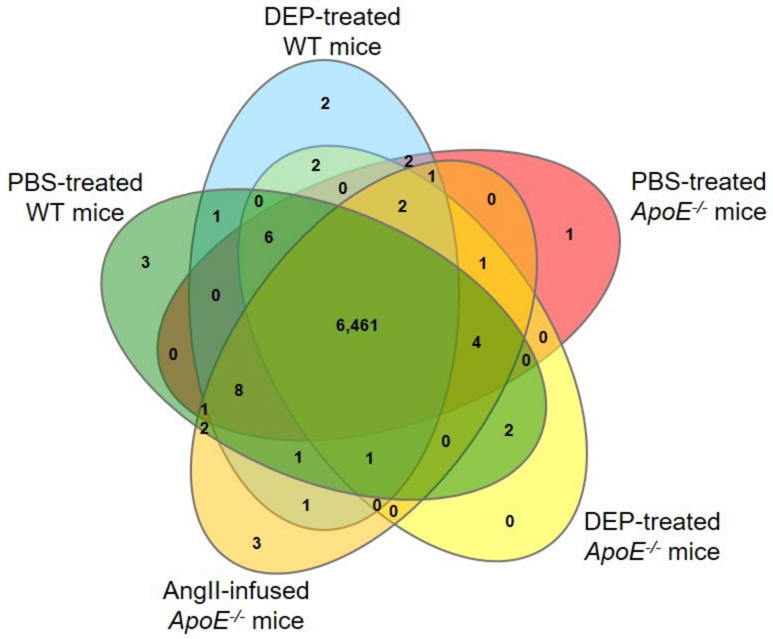

Figure S2. Venn diagrams showing the numbers of proteins identified by LC-MS/MS analysis from PBS-treated WT mice, DEP-treated WT mice, PBS-treated *ApoE*$^{-/-}$ mice, DEP-treated *ApoE*$^{-/-}$ mice, and AngII-infused *ApoE*$^{-/-}$ mice. Of the identified proteins, 6,461 proteins were commonly present in samples from the five mouse groups.

Uncropped images of western blot results

The membranes were cut prior to hybridization with antibodies; thus, the images of full-length blots cannot be provided. Area of the blots which was used in Figures were highlighted by dotted-red delineation.

As for the Figure 1D, two membranes, which were separately prepared for α-SMA and α-tubulin, were simultaneously developed for α-SMA and α-tubulin and the band images with dotted-red rectangles were finally included in Figure 1D.

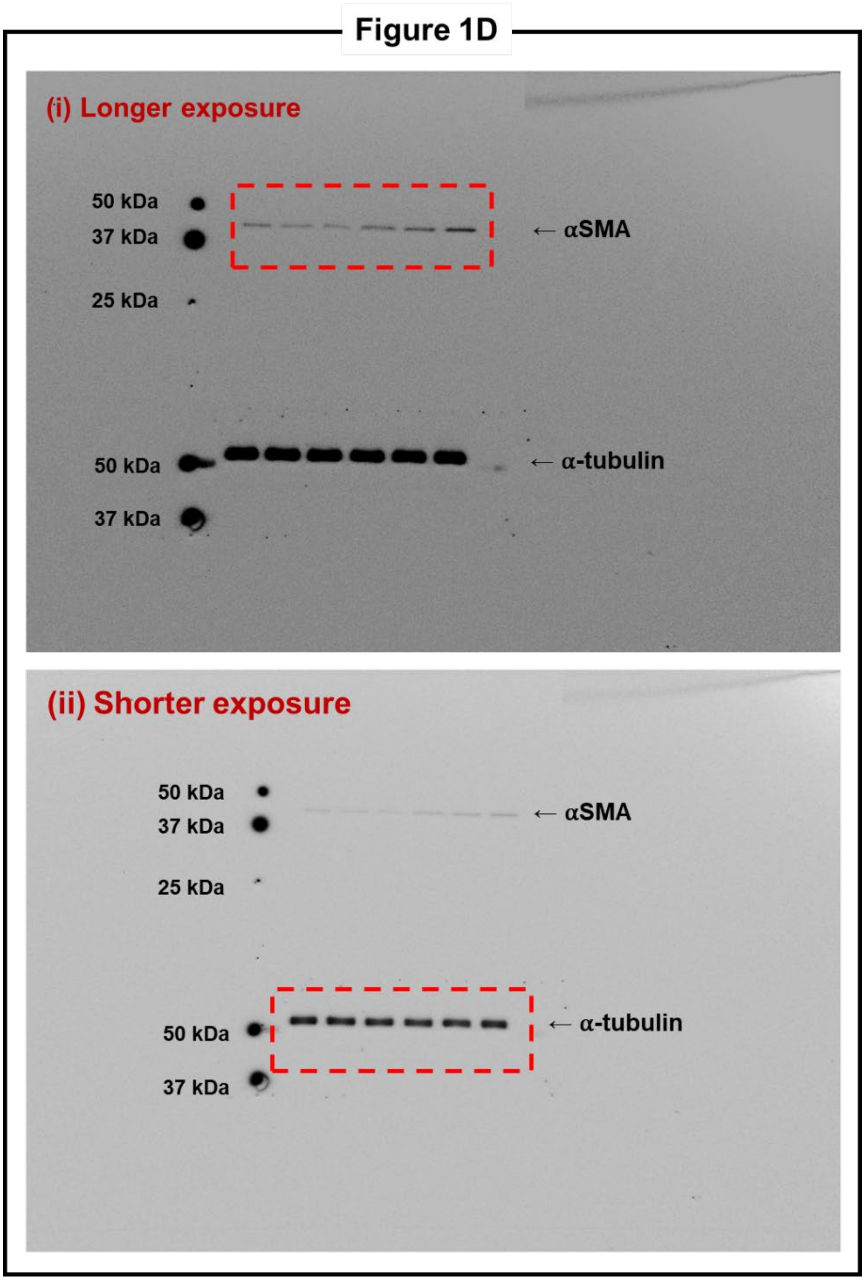

Figure 4A

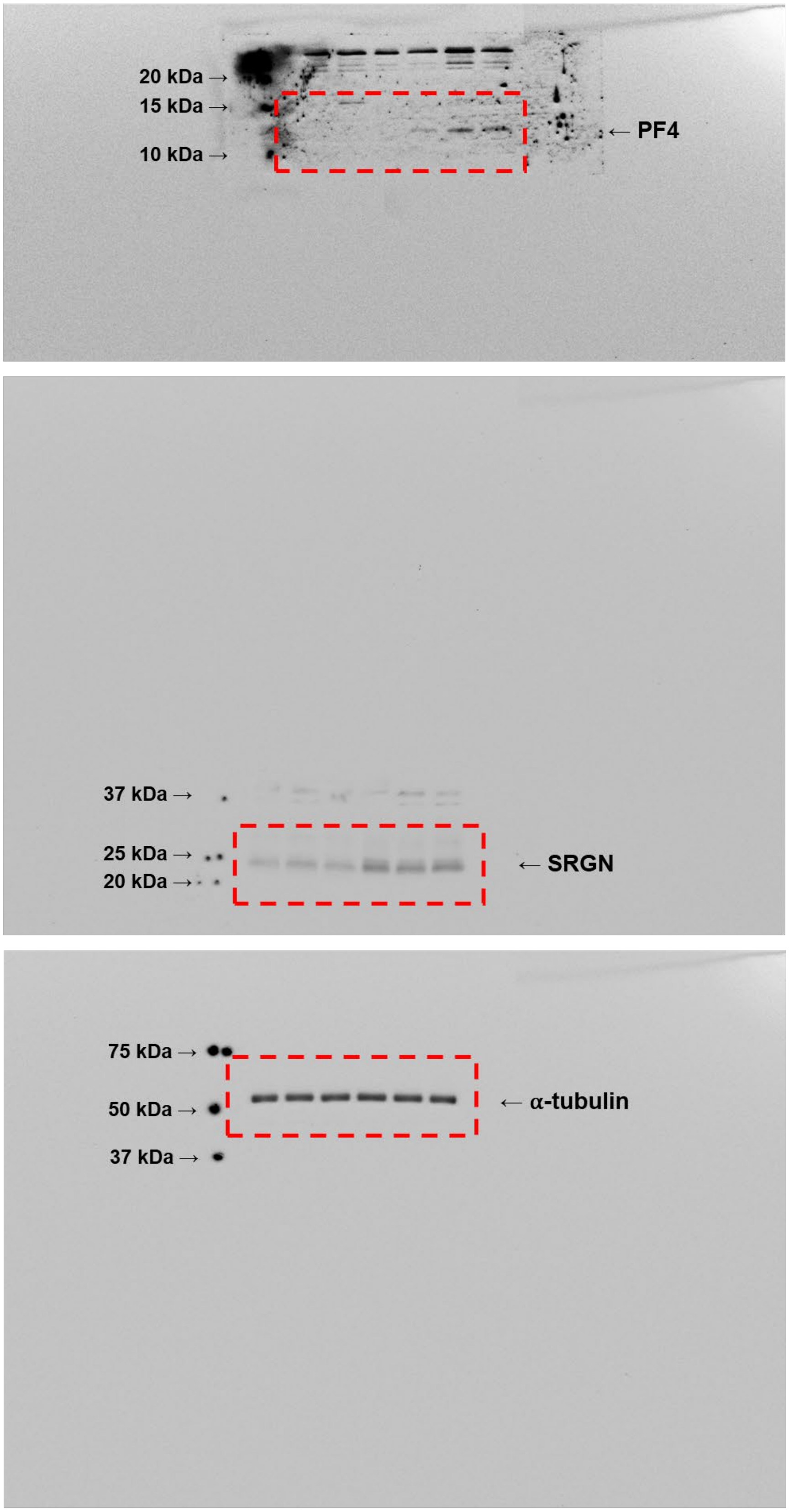
20 kDa →
15 kDa →
10 kDa →
← PF4
37 kDa →
25 kDa →
20 kDa →
← SRGN
75 kDa →
50 kDa →
37 kDa →
← α-tubulin
